# Determinants of parental seasonal influenza vaccine hesitancy in the Eastern Mediterranean region: A cross-sectional study

**DOI:** 10.3389/fpubh.2023.1132798

**Published:** 2023-03-28

**Authors:** Noha Fadl, Salah T. Al Awaidy, Abdelhamid Elshabrawy, Mona Sayed Aly Hassan Makhlouf, Sarah Assem Ibrahim, Suzan Abdel-Rahman, Nazir Ahmad Tookhy, Abdullah Alsalmani, Mays Al-Saeedi, Ibrahim Al-Sawalha, Mohammad Amin Aly El-Din, Janet Saad, Zainab Ayoob, Mohamed Khalil Rourou, Manahil Ali, Salha M. Tawati, Yahia Marwan Ahmed Gadain, Sara Yunis Al-saidi, Ghadeer Ali Hassan, Mariam Alsanafi, Leen Sandouk, Naglaa Youssef, Shaykhah Alothman, Saja Yazbek, Khlood Saleh Al-Ansi, Slimane Mehdad, Mohammed Fathelrahman Adam, Assem Gebreal, Ramy Mohamed Ghazy

**Affiliations:** ^1^Department of Family Health, High Institute of Public Health, Alexandria University, Alexandria, Egypt; ^2^Office of Health Affairs, Ministry of Health, Muscat, Oman; ^3^Middle East, Eurasia and Africa Influenza Stakeholders Network (ME’NA-ISN), Cape Town, South Africa; ^4^Faculty of Graduate Studies for Statistical Research, Cairo University, Cairo, Egypt; ^5^Department of Pediatric, Al Galaa Teaching Hospital, General Organization for Teaching Hospitals and Institutes (GOTHI), Ministry of Health and Population, Cairo, Egypt; ^6^Department of Paraclinic, Faculty of Veterinary Science, Herat University, Herat, Afghanistan; ^7^National Space Science and Technology Center, United Arab Emirates University, Al Ain, United Arab Emirates; ^8^College of Pharmacy, Al Ain University, Al Ain, United Arab Emirates; ^9^Faculty of Medicine, Jordan University of Science and Technology, Irbid, Jordan; ^10^Abou Al Monagga Central Hospital, Ministry of Health and Population, Qalyubia, Egypt; ^11^Ministry of Health, Cairo, Egypt; ^12^Faculty of Medicine, Alexandria University, Alexandria, Egypt; ^13^University of Medicine and Pharmacy of Iasi, Iasi, Romania; ^14^Karachi Medical and Dental College, Karachi, Pakistan; ^15^Department of Pharmaceutical Chemistry, Benghazi University, Benghazi, Libya; ^16^Faculty of Medicine, The National Ribat University, Khartoum, Sudan; ^17^Faculty of Dentistry, Alazhar University, Gaza, Palestine; ^18^Faculty of Medicine, Thi Qar University, Nasiriya, Iraq; ^19^Department of Pharmacy Practice, Faculty of Pharmacy, Kuwait University, Kuwait, Kuwait; ^20^Faculty of Pharmacy, Arab International University, Daraa, Syria; ^21^Department of Medical-Surgical Nursing, College of Nursing, Princess Nourah Bint Abdulrahman University, Riyadh, Saudi Arabia; ^22^Organ Transplant Pediatric Clinic, King Faisal Specialist Hospital and Research Center, Riyadh, Saudi Arabia; ^23^Faculty of Public Health, Lebanese University, Beirut, Lebanon; ^24^21 September University of Medical and Applied Science, Yemen, Yemen; ^25^Physiology and Physiopathology Research Team, Research Centre of Human Pathology Genomics, Faculty of Sciences, Mohammed V University in Rabat, Rabat, Morocco; ^26^Faculty of Pharmacy, University of Science and Technology, Omdurman, Sudan; ^27^Alexandria Faculty of Medicine, Alexandria University, Alexandria, Egypt; ^28^Tropical Health Department, High Institute of Public Health, Alexandria University, Alexandria, Egypt

**Keywords:** influenza, vaccination, parental hesitancy, parental attitude, childhood, Eastern Mediterranean

## Abstract

**Background:**

Seasonal influenza vaccine can reduce the risk of influenza-associated hospitalizations and deaths among children. Given that parents are the primary decision makers, this study examined the parental attitude toward childhood influenza vaccine and identified determinants of vaccine hesitancy (VH) in the Eastern Mediterranean region (EMR).

**Methods:**

A cross-sectional study was conducted using an anonymous online survey in 14 EMR countries. Parents of children aged 6 months to 18 years were included. The Parent Attitude about Childhood Vaccines (PACV) was used to assess VH. Chi square test and independent t-test were used to test for association of qualitative and quantitative variables, respectively. A structural equations model (SEM) was used to identify direct and indirect determinants of parental VH.

**Results:**

Almost half of the parents were hesitant about vaccinating their children against influenza (50.8%). Parental VH was significantly higher among older mothers (37.06 ± 8.8 years, *p* = 0.006), rural residents (53.6%, *p* < 0.001), high-income countries residents (50.6%, *p* < 0.001), and mothers with higher educational levels (52.1%, *p* < 0.001). Parents of school-aged children (5–9 years) (55.6%, *p* < 0.001), children free from any comorbidities (52.5%, *p* < 0.001), children who did not receive routine vaccination at all (51.5%, *p* = 0.03), children who were not vaccinated against COVID-19 (54.3%, *p* < 0.001), in addition to parents who were not vaccinated against influenza (57.1%, *p* < 0.001) were significantly associated with increased likelihood of VH. Parents who were depending on healthcare provider as a source of information regarding vaccines were less likely to report VH (47.9%, *p* < 0.001), meanwhile those who used social media as their source of health information showed a significantly higher VH (57.2%, *p* < 0.001). The SEM suggested that mother’s age, residence, country income level, child gender, total number of children and source of information regarding vaccines had a direct effect on VH. Meanwhile, parents vaccinated against influenza, children completely or partially vaccinated with routine vaccines and children vaccinated against Coronavirus disease 2019 (COVID-19) had an indirect effect on VH.

**Conclusion:**

A high proportion of included parents were hesitant to vaccinate their children against seasonal influenza. This attitude is due to many modifiable and non-modifiable factors that can be targeted to improve vaccination coverage.

## 1. Introduction

Seasonal influenza is an acute respiratory illness caused by influenza viruses. Seasonal influenza viruses come in four different types: A, B, C, and D. The influenza A and B viruses result in yearly epidemics. Pandemics have only been reported to be caused by influenza type A viruses. The influenza C virus is less often seen, it typically causes minor illnesses. Influenza D viruses are known to mainly infect cattle; they are not known to infect humans (1). According to the World Health Organization (WHO), the influenza epidemics result in between 290,000 and 650,000 reported global mortality, 3–5 million severe cases with secondary complications annually, which are most prevalent among the elderly, patients with comorbidities, infants and toddlers (2). The Eastern Mediterranean Region (EMR) is a home to 22 countries and is a major area for the spread of influenza, with winter (December to March) being the period when the virus is at its most active state. However, some countries like Qatar and Oman have a two-peak pattern every year that resembles the tropics (3).

Seasonal influenza vaccination is the most effective intervention to reduce the incidence of influenza and mitigate disease severity. The Centers for Disease Control and Prevention (CDC) recommends annual influenza vaccination for all children older than 6 months and who do not have contraindications (4). Unfortunately, the share of seasonal influenza vaccination in the EMR is approximately 2.2% of the globally distributed doses (5).

Although seasonal influenza infection is linked to severe morbidity and mortality, especially in young children, seasonal influenza vaccination is one of the vaccines that is associated with many debates among parents (6). Due to declining vaccination rates and the rise of anti-vaccination campaigns, the world is currently dealing with a number of outbreaks of infectious diseases that represent a threat to children’s health and can be prevented by vaccination (7). The anti-vaccine attitude has been extended to low- and middle-income countries (LMICs) of the EMR, specially against coronavirus disease 2019 (COVID-19) among adults (8–10), and parents (11, 12).

Vaccine hesitancy (VH) is defined by the WHO as a delay in acceptance or refusal of vaccines in spite of the availability of vaccination services (13). The Vaccine Hesitancy Matrix was created by the WHO (14), which broadly divides the causative factors of hesitancy into “contextual influences,” “individual and group influences,” and “vaccine- or vaccination-specific issues” and offers a comprehensive framework for understanding VH. By employing open-ended questions for these causal factors, the Strategic Advisory Group of Experts (SAGE) established indicators for VH according to the national schedule (15).

In the EMR, parental VH toward routine immunization and COVID-19 has been investigated, but limited studies specifically addressed the influenza VH (12, 16–18). In the United Arabs of Emirates (UAE), only 12% of the caregivers were hesitant to give their children routine vaccines (17). Meanwhile, in Egypt and Saudi Arabia, 70.6 and 61.9% of the parents hesitated to vaccinate their children against COVID-19, respectively (12, 16). Interestingly, high VH toward influenza was observed among Jordanian health care workers (18). Such a study could assist in the development of evidence-based interventions to improve influenza vaccine equity and optimize vaccine uptake among children. Thus, the aim of the present study was to investigate parental attitude toward childhood seasonal influenza vaccine and identify determinants of VH using the validated Parent Attitude about Childhood vaccines (PACV).

## 2. Methods

### 2.1. Study design and study participants

A cross-sectional study was conducted using an online survey (Qualtrics platform and Google form). Parents/guardians of children aged 6 months to 18 years were recruited using a convenient snowball sampling method from 14 EMR countries; Afghanistan, Bahrain, Egypt, Iraq, Jordan, Kuwait, Libya, Palestine, Pakistan, Qatar, Saudi Arabia, Syria, Tunisia, and UAE. The youngest child was included when parents had more than one child in the target age group. Parents of children under 6 months were excluded because the influenza vaccine is not recommended for this group. The online survey was distributed in bilingual format (Arabic and English) *via* social media channels to reach out most of the participants. The survey was administered from September 8th to October 16th, 2022.

**Figure 1:**
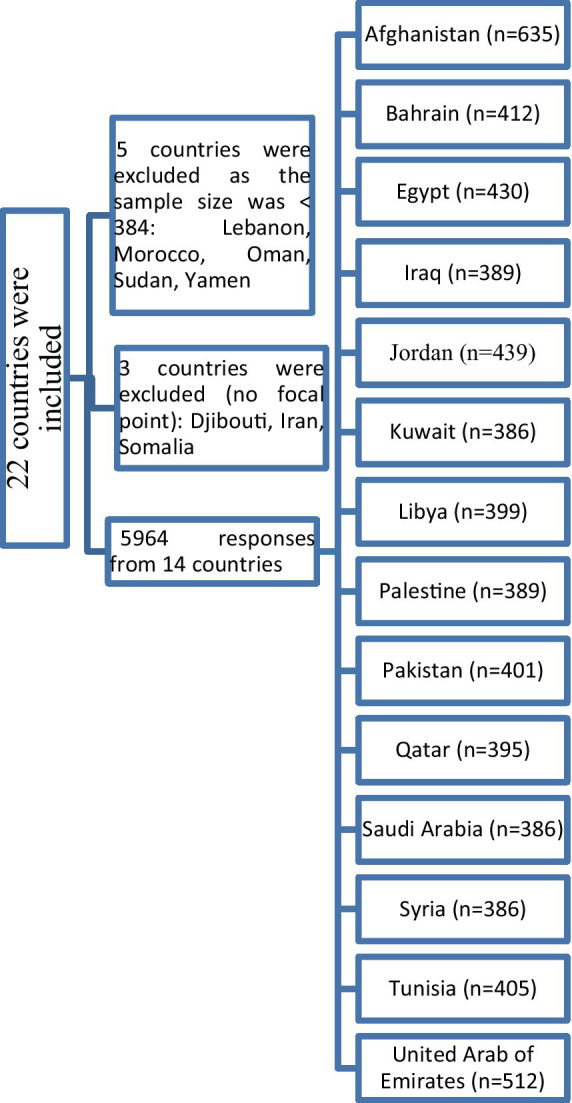
Flowchart of the study sample distribution across the Eastern Mediterranean Region.

Since the main language used in Pakistan is Urdu and the main languages used in Afghanistan are Pashto and Persian and there are no validated versions of the PACV in these languages, data collectors distributed the survey through online platforms in addition to face-to-face interviews to collect the required sample size. Based on an assumption of a prevalence rate of 50% for parental VH toward their children influenza vaccination, using 5% accepted degree of precision, α of 0.05 and power of 80%, the minimal required sample size was 384 participants from each country. The total sample size for the included countries was 5,376 and it was increased to 6,000 to compensate for missing data. The distribution of study sample size across the EMR is shown in Figure 1. We excluded 36 responses as they had incomplete data. The sample size was calculated using EPI-Info software version 7.2.5.

### 2.2. Data collection

Data collection sheet was constructed based on literature review (13, 19, 20). Sociodemographic data was collected including country, residence area, mother’s age, mother’s education, mother’s occupation, total number of children (Supplementary File S1). Countries were classified according to the World Bank into 4 categories; high-income (Bahrain, Kuwait, Qatar, Saudi Arabia and UAE), upper middle income (Iraq, Jordan and Libya), lower middle income (Egypt, Palestine, Pakistan and Tunisia), and low income countries (Afghanistan and Syria) (21). Parental influenza vaccine uptake and source of information on vaccines were added. Data about children such as age, gender, birth order, and presence of chronic comorbidity were collected. Age of children were categorized into infants: from 6 months to the first year of life, preschool age: 2–4 years, school age: 5–9 years, and adolescents: 10–18 years. Childhood routine vaccination uptake and COVID-19 vaccine uptake were determined based on parental recall.

The English and Arabic validated versions of the PACV were used to identify parents who were hesitant toward childhood influenza vaccine (22, 23). The PACV has 15 elements with three domains: behavior toward the vaccine, beliefs about the safety and efficacy of the vaccine, and general attitudes and trust. The PACV response formats (dichotomous, 5-point Likert scale and 10-point Likert scale) were collapsed into three response categories: ‘Hesitant’ responses receive a score of 2, ‘not sure or do not know’ a score of 1, and ‘not hesitant’ 0. The total PACV score was calculated by summing each item and transformed into a 0–100 scale. The total PACV score generated, with a score < 50 indicating non hesitancy and ≥ 50 indicating hesitancy (23). The overall Cronbach’s alpha value of the PACV was 0.726.

A pilot test of the questionnaire was carried out to test its applicability. Corrective measures were taken including linguistic corrections to make sure that the language was clear. Additionally, a question asking about income was removed as the majority of pilot-test participants reported that it was a sensitive question. Pilot-test participants were excluded from the final study sample.

### 2.3. Statistical analysis

The frequency distribution and descriptive statistics (mean ± standard deviation) were used to describe the characteristics of the study participants. Bivariate analysis using the Chi-square test was performed to analyze the study participants according to hesitant or non-hesitant to vaccination. The Exact Fisher test or Monte Carlo test was used when Chi-square test assumptions were violated. Independent t-test was used to compare the means of quantitative variables between two unrelated groups. Two-tailed *p* value <0.05 was considered statistically significant. Statistical testing was performed using the Statistical Package for Social Sciences (SPSS), version 22.0 (IBM Corp., Armonk, NY, United States). A structural equations model (SEM) using the Analysis of Moment Structures (AMOS) software (version 23), was built to assess the direct, indirect, and total effects of exogenous and endogenous variables on the PACV. The goodness of fit was investigated using the root mean square residual (RMSR), the goodness of fit index (GFI) >0.9, comparative fit index (CFI) >0.9, and the normed fit index >0.9.

### 2.4. Ethical approval

This study was approved by the Alexandria Faculty of Medicine Ethics’ Committee (IRB number 00012098/0305688). After explanation the study purpose, all participants agreed to the online informed consent before filling out the questionnaire. The online survey was conducted anonymously to ensure the confidentiality of data.

## 3. Results

### 3.1. Characteristics of study participants

The mean age of the mothers was 36.7 ± 9.1 years, more than three-quarters of them were high school graduates or higher (4,725, 79.2%), and nearly one-fifth were low-income country residents (1,021, 17.1%). Children of the participants were nearly equally distributed by sex [male (3,085, 51.7%) vs. female (2,879, 48.3%)], with age group classified into four categories: infants (1,317, 22.1%), preschool children (1,647, 27.6%), school children (1,420, 23.8%), and adolescents (1,580, 26.5%). Other characteristics of the study participants are demonstrated in Table 1.

**Table 1:** Characteristics of study participants by parental attitudes toward influenza vaccine.

Variables	Overall (*N* = 5,964)	Parental attitude toward influenza vaccine		*p* value
	*n* (%)	Hesitant 3,018 (50.6)	Not hesitant 2,946 (49.4)	
Mother’s age, years				0.006*
Mean ± SD	36.7 ± 9.1	37.06 ± 8.8	36.41 ± 9.4	
Residence				
Urban	4,988 (83.6)	2,534 (50.8)	2,454 (49.2)	0.001*
Rural	795 (13.3)	426 (53.6)	369 (46.4)	
Mountains. Desert	181 (3.0)	58 (32.0)	123 (68.0)	
Country based on World Bank				
High income	2,091 (35.1)	1,058 (50.6)	1,033 (49.4)	0.001*
Upper middle-income	1,227 (20.6)	748 (61.0)	479 (39.0)	
Lower middle-income	1,625 (27.2)	814 (50.1)	811 (49.9)	
Low income	1,021 (17.1)	398 (39.0)	623 (61.0)	
Mother education				
Not educated	348 (5.8)	158 (45.4)	190 (54.6)	0.001 *
Less than high school	891 (14.9)	398 (44.7)	493 (55.3)	
High school/University/Postgraduate	4,725 (79.2)	2,462 (52.1)	2,263 (47.9)	
Mother occupation				
Working	2,913 (48.8)	1,485 (51.0)	1,428 (49.0)	0.295
Not employed	3,051 (51.2)	1,533 (50.2)	1,518 (49.8)	
Child sex				
Male	3,085 (51.7)	1,574 (51.0)	1,511 (49.0)	0.261
Female	2,879 (48.3)	1,444 (50.2)	1,435 (49.8)	
Child age				
6 months–1 Year	1,317 (22.1)	667 (50.6)	650 (49.4)	0.001*
2 years–4 years	1,647 (27.6)	807 (49.0)	840 (51.0)	
5 years–9 years	1,420 (23.8)	789 (55.6)	631 (44.4)	
10 years–18 years	1,580 (26.5)	755 (47.8)	825 (52.2)	
Birth order of the child				
Mean ± SD	3.23 ± 2.04	3.23 ± 1.9	3.23 ± 2.2	0.992
Total number of children				
Mean ± SD	3.22 ± 1.9	3.22 ± 1.8	3.21 ± 1.9	0.804
Child with chronic illness				
No	4,969 (83.3%)	2,610 (52.5)	2,359 (47.5)	0.001*
Yes	995 (16.7%)	408 (41.0)	587 (59)	
Child received the routine vaccines				
Completely vaccinated	4,484 (75.2)	2,305 (51.4)	2,179 (48.6)	0.030*
Partially vaccinated	1,092 (18.3)	513 (47.0)	579 (53.0)	
Not vaccinated	388 (6.5)	200 (51.5)	188 (48.5)	
Child received the COVID-19 vaccine				
No	4,597 (22.9)	2,497 (54.3)	2,100 (45.7)	0.001*
Yes	1,367 (77.1)	521 (38.1)	846 (61.9)	
Parents received the influenza vaccine				
No	3,786 (63.5)	2,162 (57.1)	1,624 (42.9)	0.001*
Yes	2,178 (36.5)	856 (39.3)	1,322 (60.7)	
Source of information regarding vaccines				
Healthcare provider (Doctor or nurse)				
No	3,786 (63.5)	962 (57.5)	711 (42.5)	0.001*
Yes	2,178 (36.5)	2,056 (47.9)	2,236 (52.1)	
Family and friends				
No	3,907 (65.5)	1,964 (50.3)	1,943 (49.7)	0.246
Yes	2,057 (34.5)	1,054 (51.2)	1,003 (48.8)	
School				
No	4,746 (79.6)	2,389 (50.3)	2,357 (49.7)	0.218
Yes	1,218 (20.6)	629 (51.6)	589 (48.4)	
Social media and internet				
No	3,878 (65.0)	1,824 (47.0)	2,054 (53.0)	0.001*
Yes	2,086 (35.0)	1,194 (57.2)	892 (42.8)	
TV programs				
No	4,789 (80.3)	2,417 (50.5)	2,372 (49.5)	0.350
Yes	1,175 (19.7)	601 (51.1)	574 (48.9)	
Other sources (journals, scientific papers)				
No	5,306 (89.0)	2,668 (50.3)	2,638 (49.7)	0.086
Yes	731 (11.0)	350 (53.2)	308 (46.8)	

*Significant.

### 3.2. Parental attitude toward influenza vaccine and its determinants

Nearly half of the parents were hesitant about vaccinating their children against seasonal influenza (3,018, 50.6%). Parental VH was significantly higher among older mothers (37.06 ± 8.8 years, *p* = 0.006), rural residents (426, 53.6%, *p* < 0.001), high-income countries residents (1,058, 50.6%, *p* < 0.001), and mothers with higher educational levels (2,462, 52.1%, *p* < 0.001), children free from any comorbidities (2,610, 52.5%, *p* < 0.001), children not getting routine vaccination at all (200, 51.5%, *p* = 0.03), children not vaccinated against COVID-19 (2,497, 54.3%, *p* < 0.001), in addition to parents who were not vaccinated against seasonal influenza (2,162, 57.1%, *p* < 0.001) were significantly associated with likelihood of VH. Parents depending on healthcare provider as a source of information regarding vaccines were less likely to report VH (2056, 47.9%, *p* < 0.001), meanwhile those who used social media as their source showed a significantly higher VH (1,194, 57.2%, *p* < 0.001; Table 1).

### 3.3. Structural equation model

**Figure 2:**
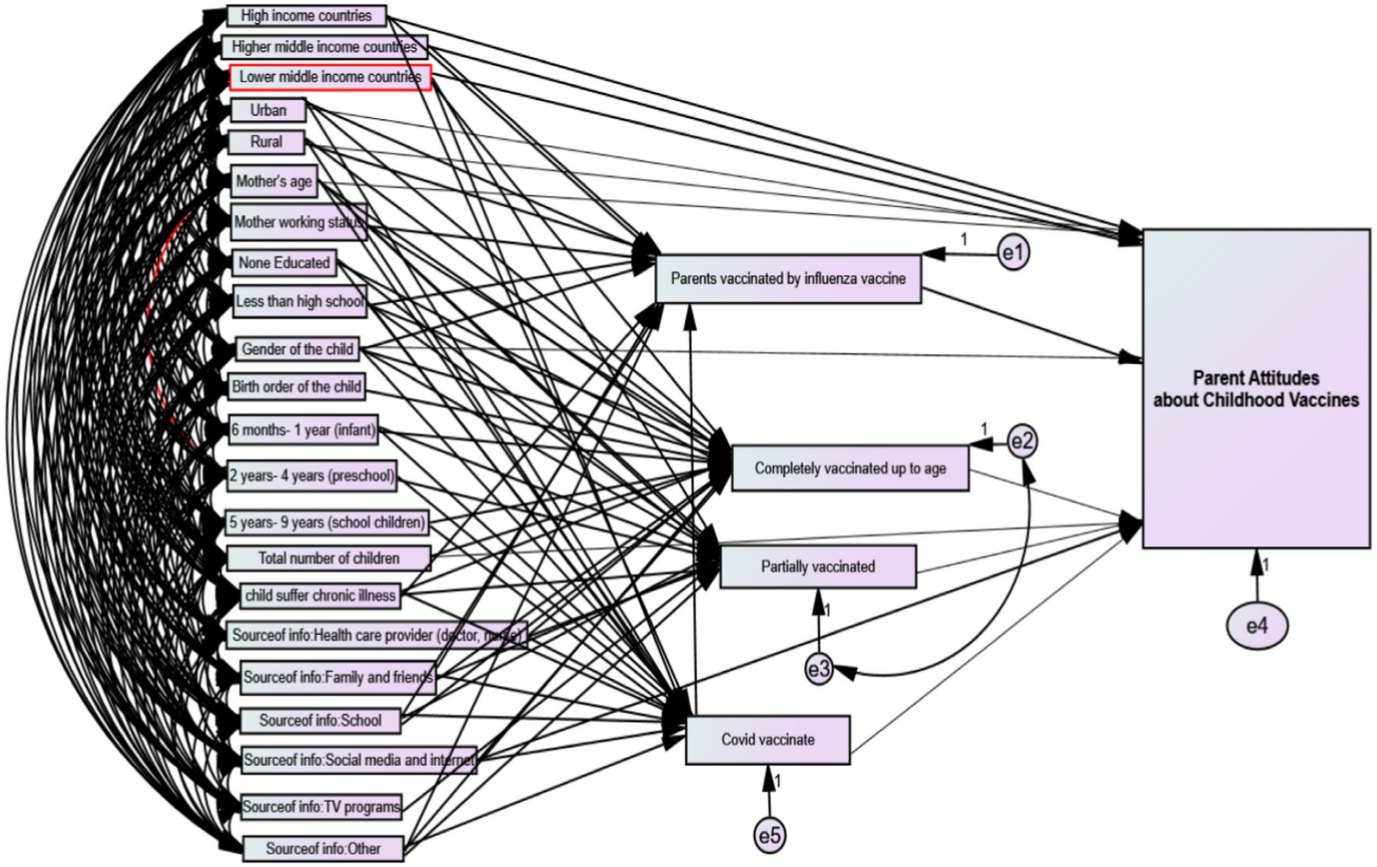
Visualization of the structural equations model for parental attitude toward childhood influenza vaccines.

Figure 2 shows the total, direct, and indirect effect of exogenous and endogenous variables on the parental attitude toward childhood seasonal influenza vaccines using the SEM. Supplementary Table S1 shows the variables included in the SEM. The framework suggests that exogenous variables: mother’s age, residence (urban, rural), country income level (high-income countries, upper middle-income countries, lower middle-income countries), gender of the child, total number of children, and source of information regarding vaccines (social media and internet) had a direct effect on PACV. High-income country residency had a positive total effect on parental attitude toward vaccination, this means that parents living in high-income countries were more likely to be hesitant regarding vaccinating their children compared to those from low-income countries by positive total effect value of 4.03. The total, direct and indirect effects of other exogenous variables are demonstrated in Table 2.

**Table 2:** Value and direction of the total, direct, and indirect effect of exogenous variables on parental attitude toward childhood influenza vaccines.

Variable	Total effect and direction	Direct effect and direction	Indirect effect and direction
High income countries	4.03	5.76	−1.73
Upper middle-income countries	6.78	7.17	−0.39
Lower middle-income countries	2.36	2.85	−0.48
Urban residence	2.07	1.93	0.14
Rural residence	5.50	4.85	0.65
Mother’s age	0.07	0.11	−0.04
Mother occupation	0.48	0.000	0.48
Not Educated	−0.02	0.000	−0.02
Less than high school	0.26	0.000	0.26
Child gender	−0.36	−0.98	0.62
Birth order of the child	0.05	0.000	0.05
Child age: 6 months- 1 year	1.74	0.000	1.74
Child age: 2 years- 4 years	1.87	0.000	1.87
Child age: 5 years- 9 years	1.93	0.000	1.93
Total number of children	0.27	0.25	0.02
children with chronic illness	1.47	0.000	1.47
Source of information: Health care provider (doctor, nurse)	0.35	0.000	0.35
Source of information: Family and friends	−0.29	0.000	−0.29
Source of information: School	1.02	0.000	1.02
Source of information: social media and internet	−3.34	−3.22	−0.11
Source of information: TV programs	0.05	0.000	0.05
Source of information: Other	−1.02	0.000	−1.02

Table 3 shows the total, direct and indirect effects of the endogenous variables on parental attitude toward childhood seasonal influenza vaccines. Parents vaccinated against seasonal influenza had a positive total and direct effect on PACV with value of 6.74, while children completely and partially vaccinated have a negative total and direct effect on PACV with value of −4.57 and −3.76, respectively. There is no indirect effect of parents vaccinated against seasonal influenza, children completely or partially vaccinated on PACV; meanwhile children vaccinated against COVID-19 had an indirect effect on PACV with value of 1.67.

**Table 3:** Value and direction of the total, direct, and indirect effect of endogenous (intermediate) variables on parental attitude toward childhood influenza vaccines.

Variable	Total effect and direction	Direct effect and direction	Indirect effect and direction
Parents received the influenza vaccines	6.74	6.74	0.000
Child received the routine vaccines completely up to age	−4.57	−4.57	0.000
Child received the routine vaccines partially	−3.76	−3.76	0.000
Child received the COVID vaccines	6.06	4.39	1.67

The model had an acceptable goodness of fit (GFI = 0.998, CFI = 0.997). The minimum discrepancy index = 4.328, the root means square residual = 0.081, the goodness of fit index = 0.998, the adjusted goodness of fit index was 0.980 which is acceptable, the comparative fit index was 0.997. The normed fit index was 0.996. The parsimony ratio index was 12.8%. Finally, the root mean square error of approximation was 0.024 (Supplementary Table S2).

## 4. Discussion

In the current study, we provide the first estimates of parental attitude toward seasonal influenza vaccination from a large sample of parents in the EMR in addition to using the SEM to identify the direct and indirect determinants of VH. Almost half of the parents were hesitant to vaccinate their children with the seasonal influenza vaccine. This may be because the seasonal influenza vaccine is still a category B vaccine (non-EPI) compared to the free vaccine in the Expanded Program on Immunization (EPI) for children in most countries of the EMR. Only five EMR countries reported incorporating the influenza vaccine into their national immunization program namely; Iran, Libya, Qatar, Syria and Tunisia (5). Worldwide, the prevalence of VH among parents and caregivers toward childhood influenza vaccination ranged from 13% in Italy (33/255) (24), 25.8% in United States (530/2,176) (25), up to 56.03% in China (3,736/6,668) (19). These discrepancies among studies are likely the result of the difference in economic factors, cultural norms, level of knowledge about seasonal influenza vaccine, access to medical care, parents’ perception in addition to difference in study design and sample size.

Although seasonal influenza vaccine distribution is low in LMICs (26), the current study showed that VH was significantly higher among higher income parents. Previous studies reported that higher household incomes were negatively associated with the intention to vaccinate children against seasonal influenza (19, 27). In United States, it was also found that children living in high-affluent neighborhoods had 1.08% lower routine vaccination coverage than children in low-affluent areas (28). Furthermore, individuals in high-income countries showed more hesitancy toward COVID-19 vaccine due to concerns about vaccine safety (29). Meanwhile Harapan et al. (30) showed greater VH against COVID-19 vaccine in LMICs when compared to high income countries. On the contrary, a systematic review stated that the influence of income on seasonal influenza immunization has been inconsistent (31). The current study also showed a higher rate of VH among rural residents. Similarly, Vasudevan et al. (32) reported that rural mothers had more vaccine-related concerns compared to urban mothers. However, Lai et al. (19) reported that urban residents were more hesitant to vaccinate children. The current findings would be explained as high wealth may indicate more knowledge about medical exemptions and anti-vaccination campaigns (28), and more confidence in the quality of care provided to those of high economic status (27). Moreover, rural residence may reflect limited utilization of health care services (33), lower access to health information (34), and lower prevalence of health-related behaviors (35).

Surprisingly, the current study identified a higher educational level of mothers as a potential barrier to childhood seasonal influenza vaccination. The association between mother’s education and VH is still inconsistent; with some studies aligned with the current findings (19, 36, 37), while others found no relation (38, 39). Goldman et al. (40) suggested that parents with higher education might be less likely to change their mind about vaccination or could be more exposed to misinformation on social media. Role of education in VH is complex and may interplay with other factors influencing vaccination adherence. Furthermore, older age mothers were found to report a statistically significant impact on their VH behavior which was similar to the findings of Hamada et al. (41)

In the present study, parents of school-aged children were more likely to have VH. Similarly, a study found that parents of children aged 7–12 years (OR: 1.923), 12–14 years (OR: 2.372) had higher seasonal influenza VH compared to the under-3 years age group (36). Gates et al. (42) found that seasonal influenza vaccination coverage among children aged 5–12 years was 33.2%, which was lower than the rate reported among under-five children (52.6%). The result may be due to the fact that some parents assume that the risk of seasonal influenza infection and health threat is low and that their children’s immunity may improve with age. The current study also reported that parents of chronically ill children were less likely to show VH. Children with comorbidities are at higher risk for severe influenza and poorer progression (43), which explains why such parents would be more cautious of getting their children sick with influenza. On the contrary, Napolitano et al. (44) reported that existence of chronic medical conditions negatively affects immunization coverage rates among children, including seasonal influenza vaccination. While Almalki et al. (18) did not find an association between VH and chronic illness in children (16).

The current study showed that parents who were vaccinated against seasonal influenza showed lower level of VH. Awad et al. (45), and Alolayan et al. (38) also reported similar findings. Likewise, a previous systematic review has identified past vaccination experience and behavior as strong predictors of seasonal influenza vaccine acceptance (20). This may be because parents obtained the protective effect from the vaccine. Furthermore, the current study showed that parents of children who did not receive their EPI vaccines were more likely to have higher VH. Zakhour et al. (6) stated that parents who accepted seasonal influenza vaccination were more likely to believe in vaccine efficacy, have higher trust and show less VH and were more compliant with other vaccinations. Similarly. Goldman et al. (40) found that parents vaccinating their children up to date showed higher willingness to vaccinate them against seasonal influenza in the next year. Correspondingly, the current study found that parents whose children received the COVID-19 vaccine had a lower likelihood of VH, which is consistent with previous studies (36, 40, 46). This could reflect higher parental health literacy.

The current study showed that when a healthcare provider was the source of information about vaccines, parents were less likely to have VH. In line with present finding, Fan et al. (36) found that recommendations from medical staff to vaccinate children against seasonal influenza had reduced parental VH. Trusting in healthcare providers’ advice about seasonal influenza vaccine was also associated with decreased parental VH or refusal (19). Healthcare providers are the most trusted influencer of vaccination decisions (47). The current study also found that the use of social media as source of health information about vaccines increased the likelihood of VH. Likewise, using social media was negatively associated with seasonal influenza vaccine uptake among both pediatric (36) and adult population (48). However, Ahmed et al. (49) found that adult users of Twitter and Facebook as sources of health information were more likely to be vaccinated against influenza than those who did not use social media. Social media and internet are double-edged sword. Positive information could improve knowledge and trust, but misinformation advocating against vaccines have the opposite effect.

The current study showed that VH was not influenced by child gender, which was supported by findings of Lai et al. (19) On the contrary, a study reported that VH was significantly more common among parents who had girls (44). Additionally, the current study did not find an association between the number of children in the household and VH, which was similar to previous studies (17, 25, 44). However, another study reported that influenza VH was higher among caregivers with the only child in the family (19). Birth order of the index child did not have a significant impact on VH in the current study. Alsuwaidi et al. reported similar results (17).

### 4.1. Strength and limitations

To our knowledge, this is the first study to assess parental attitude toward seasonal influenza vaccination in the EMR. We used two languages (English and Arabic) to increase the response rate and the representation of the studied population. Another point of strength was that the analyzed data was from a large representative sample of parents in the EMR and were collected by a valid and reliable methodology. The large sample size provided the study with good statistical power. The data set also contained several contributing factors for the risk of VH including parental and child sociodemographic characteristic, child health status, and vaccination status. Furthermore, the CDC recommends offering influenza vaccine during September or October (4), that corresponds with timing of the present study and could affect parent’s decision to accept the seasonal influenza.

However, the findings of the current study should be interpreted in light of the following limitations. The online survey was susceptible to recall and social desirability bias, since it is based on self-reporting. Parental recall of their children’s vaccination status may also lead to recall bias, underreporting, and/or selective reporting. The sample selection was based on a non-probability technique using an online survey that may violate the generalization of the present findings. Another limitation was that the PACV was not validate in Urdu, Pashto and Persian languages. Further research is also needed to include more factors that could contribute to the likelihood of VH, such as the availability and accessibility of health care services and vaccination for children and other sociodemographic characteristics (e.g., fathers’ education and marital status). Finally, the inherent limitation of cross-sectional survey like recall bias and inability to assess causality could not be avoided.

## 5. Conclusion

The prevalence of parental VH toward childhood seasonal influenza vaccine in the EMR was considerably high, particularly among older mothers, residents of rural areas and high-income countries, mothers with higher educational levels, parents of school-aged children, children free from any comorbidities, children who did not receive routine vaccination at all, children not vaccinated against COVID-19, in addition to parents who were not vaccinated against influenza. The present findings have highlighted a significant public health concern regarding seasonal influenza vaccination. Understanding parental attitudes toward vaccinating their children is crucial in developing and implementing interventions to increase vaccination coverage. Moreover, plans and strategies should be directed toward incorporating seasonal influenza vaccination into the existing EPI schedules to reduce the disease burden among children in the EMR.

## Data availability statement

The raw data supporting the conclusions of this article will be made available by the authors, without undue reservation.

## Ethics statement

The studies involving human participants were reviewed and approved by the Alexandria Faculty of Medicine Ethics’ Committee IRB number (00012098/0305688). The patients/participants provided their written informed consent to participate in this study.

## Author contributions

RG and NF: conceptualization and methodology. NT, AA, MA-S, IA-S, ME-D, JS, ZA, MR, ManA, ST, YG, SA-s, GH, MarA, LS, NY, SA, SY, KA-A, SM, and MFA: data collection and data cleaning. RG, AE, SA-R, and SI: data analysis. RG, NF, AE, SA-R, and SI: data curation. RG, NF, and MM: writing–original draft preparation. RG, NF, AG, and STA: writing–review and editing. RG: visualization. All authors have read and agreed to the published version of the manuscript.

## Conflict of interest

The authors declare that the research was conducted in the absence of any commercial or financial relationships that could be construed as a potential conflict of interest.

## Publisher’s note

All claims expressed in this article are solely those of the authors and do not necessarily represent those of their affiliated organizations, or those of the publisher, the editors and the reviewers. Any product that may be evaluated in this article, or claim that may be made by its manufacturer, is not guaranteed or endorsed by the publisher.

## Abbreviations

VH: Vaccine hesitancy
PACV: Parent attitude about childhood vaccines
COVID-19: Coronavirus disease 2019
EMR: East Meditteranian Region
LMICs: Low-middle income countries
UAE: United Arab Emirates
USA: United States of America
EPI: Extended program of immunization
CDC: Centers for Diseases Control and Prevention
WHO: World Health Organization
SAGE: The Strategic Advisory Group of Experts
SEM: Structural equations model
GFI: Goodness of fit index
CFI: Comparative fit index
RMSEA: Root Mean Square Error of Approximation
AMOS: The Analysis of Moment Structures software
RMSR: The root mean square residual
OR: Odds ratio
