# Supplementary material for: Determinants of parental seasonal influenza vaccine hesitancy in the Eastern Mediterranean region: A cross-sectional study
Source: Front Public Health. 2023 Mar 28;11:1132798. doi: 10.3389/fpubh.2023.1132798 (PMC10086336; doi:10.3389/fpubh.2023.1132798)
Supplement: Supplementary file 2 [file Data_Sheet_2.PDF]

Supplementary file 2

Supplementary table 1: Exogenous and endogenous variables included in the structural equations model

Exogenous variables	Endogenous variables	
	Intermediate variables	Dependent variable
High income countries	Parents received the influenza vaccines	Parent Attitudes about Childhood Vaccines
Higher middle-income countries	Child received the routine vaccines completely up to age	
Lower middle-income countries	Child received the routine vaccines partially	
Urban residence	Child received the COVID vaccines	
Rural residence		
Mother's age		
Mother occupation		
Not Educated		
Less than high school		
Child gender		
Birth order of the child		
Child age: 6 months- 1 year		
Child age: 2 years- 4 years		
Child age: 5 years- 9 years		
Total number of children		
children with chronic illness		
Source of information: Health care provider (doctor, nurse)		
Source of information: Family and friends		
Source of information: School		

Source of information: social media and internet		
Source of information: TV programs		
Source of information: Other		

Supplementary table 2: Measurements of goodness of fit of the structural equation model

Measurement	Value	Acceptable value
**Minimum discrepancy/degrees of freedom**	4.328	The lower the value below 5, the better in goodness of fit of the model
**Root mean square residual**	0.081	The closer the value to the zero, the better the goodness of fit of the model
**Goodness of fit index**	0.998	The higher the value over 0.9, the better the goodness of fit of the model
**Adjusted goodness of fit index**	0.980	The higher the value over 0.9, the better the goodness of fit of the model
**Comparative fit index**	0.997	The higher the value over 0.9, the better the goodness of fit of the model
**Normed fit index**	0.996	The higher the value over 0.9, the better the goodness of fit of the model
**Parsimony ratio index**	12.8	
**Root mean square error of approximation**	0.024	The lower the value under 0.05, the better the goodness of fit of the model
